# Fenofibrate Nanocrystal Composite Microparticles for Intestine-Specific Oral Drug Delivery System

**DOI:** 10.3390/ph12030109

**Published:** 2019-07-16

**Authors:** Bhavesh D. Kevadiya, Liang Chen, Lu Zhang, Midhun B. Thomas, Rajesh N. Davé

**Affiliations:** 1 New Jersey Center for Engineered Particulates, New Jersey Institute of Technology, Warren Street, Newark, NJ 07102, USA; 2 Pandorum Technologies Pvt Ltd, Bangalore Bioinnovation Centre, Helix Biotech Park, Electronic City Phase 1, Bangalore, Karnataka 560100, India

**Keywords:** bioavailability, fenofibrate, media milling, nanocrystals, intestine-specific delivery, composites spherical microparticles

## Abstract

Hydrophobic drug nanocrystals (NCs) manufactured by particle engineering have been extensively investigated for enhanced oral bioavailability and therapeutic effectiveness. However, there are significant drawbacks, including fast dissolution of the nanocrystals in the gastric environment, leading to physicochemical instability. To solves this issue, we developed an innovative technique that involves the encapsulation of nanocrystals in composite spherical microparticles (NCSMs). Fenofibrate (FNB) NCs (FNB-NCs) manufactured by a wet stirred media milling (WSMM) technique and an ionotropic crosslinking method were used for FNB-NC encapsulation within gastroresistant NCSMs. Various solid-state methods were used for characterizing NCSMs. The pH-sensitive NCSMs showed a site-specific release pattern at alkaline pH and nearly 0% release at low pH (gastric environment). This phenomenon was confirmed by a real-time in situ UV-imaging system known as the surface dissolution imager (SDI), which was used to monitor drug release events by measuring the color intensity and concentration gradient formation. All these results proved that our NCSM approach is an innovative idea in oral drug delivery systems, as it resolves significant challenges in the intestine-specific release of hydrophobic drugs while avoiding fast dissolution or burst release.

## 1. Introduction

Despite significant advancements in nanoparticulate drug delivery systems, oral drug administration is still preferred over parenteral routes due to ease of administration, convenience, low cost, and versatility in dosage form preparations. In addition, it also reduces the risk of cross-infection and needle-stick injuries [1,2]. Nearly 40% of commercially available drugs belong to biopharmaceutics classification system (BCS)-II candidates, which are poorly water soluble according to the BCS [3,4]. Improvements in BCS-II drug dissolution rates and stability pose a significant challenge for formulation scientists. In order to overcome this problem, several approaches have been pursued, such as nanosuspension by particle size reduction along with surface engineering, derivatization, complexation, salt formation, supercritical fluid, and the use of solid dispersions [3,4,5,6,7]. Usually, in the case of oral drug administration, the drug particles are dissolved in the stomach before they are absorbed into intestinal lumen. However, direct oral administration of a BCS-II drug nanosuspension is not suitable due to the potential for very fast dissolution of the drug in the gastric environment and reprecipitation upon neutralization in the small intestine to form larger crystals, leading to physicochemical instability. Hence, it is preferable to formulate a solid dose form that alleviates this problem and provides improved physical stability of drug particles in the GI tract [8]. This could eventually lead to poor in vivo stability, undesired dissolution kinetics, and limited bioavailability [9,10,11,12].

Recently, considerable attention has been directed towards nanocomposite formulations of BCS-II drugs for site-specific release. The site-specific release of a drug at a specific region in the intestinal lumen can bypass gastric-acid fluid to enhance therapeutic efficiency [1,9,13,14,15]. Fenofibrate (FNB) is one of the most frequently used BCS-II drugs to reduce high cholesterol and triglyceride contents in patients [16,17]. However, FNB is also known for its limited bioavailability due to its low water solubility [17,18]. We have previously reported a nanocomposite spherical microparticle oral delivery system formed by encapsulation of drug-layered silicate nanomaterials into alginate (AL) spherical microparticles [9,19]. It was previously demonstrated that AL spherical microparticles shrink at low pH and swell in alkaline pH. This unique characteristic feature was used to design site-specific release delivery systems by encapsulation of FNB nanocrystals (NCs) (FNB-NCs). This ensured that it could not be released in the acidic or harsh environment of the stomach, while it facilitated rapid disintegration and drug release in the intestinal tract [9,19].

**Figure 1:**
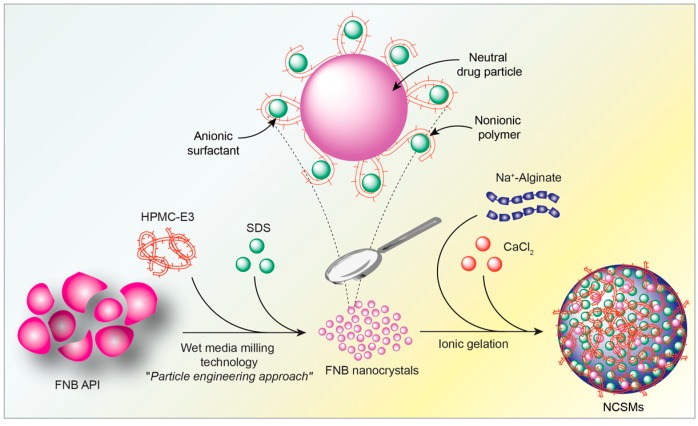
Graphical representation of the production of nanocrystals in composite spherical microparticles (NCSMs) by encapsulation of fenofibrate nanocrystals (FNB-NCs).

To this end, we developed nanocrystals in composite spherical microparticles (NCSMs) encapsulated with FNB-NCs with pH sensitivity to respond to GI tract environmental pH changes for enhanced oral drug delivery. First, highly stable aqueous FNB-NCs in a stable suspension form were created using previously established protocols for wet stirred media milling (WSMM) [17,20,21,22,23,24]. These FNB-NCs were then directly used for the development of NCSMs using an ionotropic crosslinking technique to prevent rapid gastric disintegration and site-specific release of FNB in the small intestine. An overview of NCSM production is illustrated in Figure 1. That was followed by a comprehensive characterization of the NCSMs, which included X-ray powder diffraction (XRD), thermal analysis (thermogravimetric analysis (TGA) and differential scanning calorimetry (DSC)), and attenuated total reflection Fourier-transform infrared spectroscopy (ATR-FTIR). NCSM morphology was tested using SEM, and in vitro FNB release investigations were done with an automated USP-II dissolution apparatus. Furthermore, the in situ dissolution behavior of NCSMs was characterized by a UV-based surface dissolution imager (SDI) that qualitatively recorded the dissolved drug concentration gradient in real time, depicted as color maps. Through such a multifaceted assessment, we evaluated the efficacy of NCSMs containing FNB-NCs for use as a solid dosage form for oral delivery in site-specific drug delivery applications.

## 2. Materials and Methods

### 2.1. Materials

FNB was used as a model drug and was purchased from Jay Radhe Sales (Ahmedabad, India). Hydroxypropyl methylcellulose (HPMC) (METHOCEL E3 Premium LV, Mw = ~20,300) was purchased from Dow Chemical Company (Midland, MI, USA). Sodium dodecyl sulfate (SDS) was purchased from GFS Chemicals Inc. (Columbus, OH, USA). Sodium alginate (AL) and calcium chloride were purchased from Sigma–Aldrich (St. Louis, MO, USA). All chemicals used were of analytical grade and were used as received without any modifications. Production and characterization of FNB-NCs were as per our previous study [17] and the Supplementary Materials. 

### 2.2. Preparation of NCSMs

NCSMs were prepared by modifying the solvent-free ionotropic crosslinking technique as previously used [9]. Briefly, an appropriate quantity of AL (1.0 g) was added to deionized (DI) water (50 mL) and stirred for 24 h at room temperature to get a uniform solution. The required amount of calcium chloride dihydrate was added to the DI water to make it a 5% (w/v) solution. For the preparation of NCSM formulation with various amounts of FNB-NC loading capacities, the weight ratio (w/w) of AL to FNB-NC suspension for each formulation was prepared as mentioned in Table 1. For mixing, an impeller mixer (VWR VOS 16 Overhead Stirrer, VWR International, PA, USA) operated at 300 rpm was used and stirring was maintained until the precursors of the AL spherical microparticle forming material were completely homogeneously mixed. A planetary centrifugal mixer (Thinky Mixer ARE-310, Thinky, Inc, Laguna Hills, CA, USA) was used for mixing and defoaming of the FNB-NC and AL mixture, and five different NCSM formulations were prepared using various AL:FNB-NC ratios. The AL:FNB-NC ratio (w/w) used in the formulations were: (i) 1.0:2.70 (FNB-AL-A), (ii) 1.0:6.77 (FNB-AL-B), (iii) 1.0:10.15 (FNB-AL-C), (iv) 1.0:13.55 (FNB-AL-D), and (v) 1.0:16.93 (FNB-AL-E). By dripping the obtained homogeneous suspension from an 18-gauge needle (falling distance 3.0 cm, pumping rate 2.5 mL/min) connected to a peristaltic pump (Master flex L/S 7518-00, Cole-Parmer, Vernon Hills, IL, USA), it was gradually added to 250 mL CaCl_2_ solution. The calcium chloride solution was kept over a magnetic stirrer (200 rpm) throughout the whole procedure to prevent microparticle agglomeration. In this technique, the gelled mixture retained the “spheroid shape” of the droplets, resulting in the immediate formation of NCSMs that entrapped FNB-NCs within a 3D matrix gel network. Before the NCSMs were filtered, they were restored in the CaCl_2_ solution for ~15–20 min. After filtration, the collected NCSMs were rinsed three times with DI water and dried in ambient conditions. The average diameter of the dry NCSMs was studied by measuring 50 spherical particles using a micrometer screw (Mitutoyo, Aurora, IL, USA), and the average value was used for data analysis. Placebos of AL spherical particles served as control samples. Details of formulation compositions, FNB loading, and AL:NC ratio are reported in Table 1. The effect of crosslinker concentrations on FNB-NC loading characteristics was optimized and tested with different concentrations (2.5–8% (w/v)) of CaCl_2_, while all the remaining experimental parameters were the same as stated above.

### 2.3. Determination of Drug Content

Drug quantifications were prepared by adding NCSM samples to 250 mL of SDS medium (7.2 mg/mL, pH = 8.1), stirring for 24 h, and measuring the UV absorbance at λmax = 290 nm [17]. Averaging 10 samples, quantifications of FNB were completed and the mean values are reported.

### 2.4. XRD

The solid-state polymorph of different formulations was studied using XRD (Siemens, Philips PW3040 X-ray diffractometer, Cambridge, MA, USA) to characterize all the sample. For the analysis, the following settings were used: Philips X’celerator detector and Philips X’Pert Data Collector with the curved Ni-filtered Cu-Kα (λ = 1.542 Å) radiation and scanning of 2°/min in 2θ range of 10°–40°.

### 2.5. TGA

TGA was carried out at 30–250 °C at the heating rate of 10 °C/min under nitrogen flow (20 mL/min) using the TGA/DSC STARe system (Mettler-Toledo, Inc, Columbus, OH, USA) for characterizing residual water in the nanocomposites. 

### 2.6. DSC

DSC studies were carried out in the range of 30–150 °C at the rate 10 °C/min under nitrogen flow (20 mL/min) using the Polymer DSC, TGA/DSC STARe system (Mettler-Toledo, Inc, Columbus, OH, USA). Data analysis was performed using STARe 10 software provided by Mettler-Toledo.

### 2.7. FTIR

ATR-FTIR spectra of the samples were generated by a Magna Model 560 instrument (Nicolet Instrument Corporation, Madison, WI, USA) attached to an ATR accessory with a single reflection Zn–Se crystal (MIRa-cle; Pike Technologies, Madison, WI, USA).

### 2.8. Morphology Analysis

The surface morphology of NCSMs was observed by SEM (LEO 1530 VP, New York, NY, USA). Before SEM analysis, all samples were coated with carbon using a sputter coater to enhance conductivity. NCSM microscopic images were acquired using a digital microscope (Keyence VH -2500, Illinois, IL, USA)

### 2.9. UV–Visible Spectroscopy

The UV–visible absorbance of drug solutions was measured at λ_max_ = 290 nm using a UV–visible spectrophotometer (Evolution TM 300, Thermo Scientific, Massachusetts, MA, USA) equipped with a quartz cell that had a path length of 1 cm.

### 2.10. In Vitro Drug Release Test

**Figure 2:**
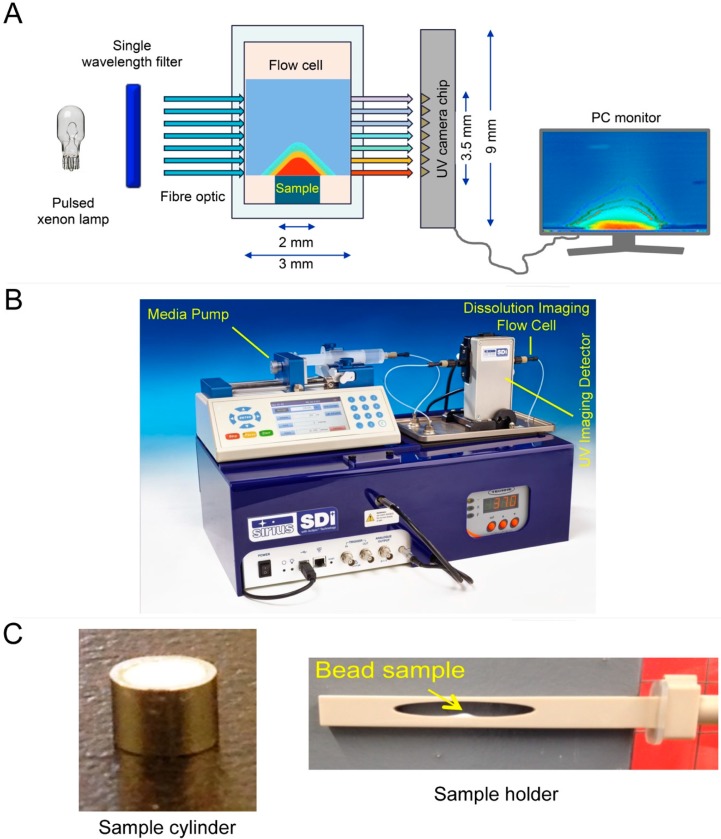
Graphical representation of the working principle of (**A**) surface dissolution imager (SDI) technology, (**B**) the instrument, and (**C**) sample preparation procedure.

In vitro release of FNB from the NCSM formulations was performed using an automated USP-II dissolution apparatus. The Sotax AT7 smart automated system (Sotax, Basel, Switzerland) contained an automatic sample collector pump and a UV–Vis spectrophotometer (Evolution Array, Thermo scientific, Philadelphia, PA, USA). Computer-controlled sample fraction collection was performed at predetermined intervals and automatic measurement of the drug was done using the software Win-Sotax, version 1.3.4 (Basel, Switzerland). The dissolution tester, with 1.0 L capacity vessels and an integrated cover, was used at 37 ± 0.1 °C. For dissolution medium, solutions of pH 1.3 (3.0 mg/mL SDS and pH adjusted with HCL) and pH 8.1 (7.2 mg/mL SDS) were used. Accurately weighed amounts of pristine FNB and NCSM formulations were placed in vessels containing 900 mL of the media. The pedal speed was set at 50 rpm. A Whatman microfiber filter (0.2 µm and 25 mm diameter) (GF/D, GE Healthcare UK limited, UK) in the top of the vessel’s opening for the collection nozzle removed the undissolved drug particles. The autosampler that collected aliquots at predetermined intervals was immediately replaced by the same medium after being spectrophotometrically analyzed at λ_max_ = 290 nm (recirculation mode). All tests were repeated at least four times and generated the mean value of the drug dissolution data with time. In vitro drug release by the UV-imaging system (surface dissolution imager (SDI)), calibration curves, and drug release were done as per a previously reported procedure [17] (Figure 2).

## 3. Results and Discussion

The objective of the present study was the site-specific controlled release of FNB. This was achieved by making stable FNB-NCs using WSMM, which were further encapsulated in NCSMs for intestinal site-specific release. Biodegradable polymeric microparticles have been preferred in pharmaceutical industries as these serve as site-specific controlled release oral drug delivery applications. Specifically, pH-responsive hydrogel spherical microparticles have been widely used to vary the pH of the medium for site-specific controlled release [19,25]. AL has special characteristic features and forms a gel in interaction with Ca^2+^ [19,25]. This gel is formed via crosslinks with the divalent ions and negatively charged carboxyl groups of the AL chain [19,25,26]. However, there are few reports in the literature on the encapsulation of pure FNB-NCs into AL microspheres or spherical particles for site-specific release as oral dosage forms without any alteration of drug NC morphology and loss of therapeutic activity of compounds while attaining higher drug-loading capacity. AL-based NCSMs are one of the site-specific delivery systems that protect drugs from the harsh stomach environment and has enhanced bioavailability on its release in the intestinal environment [19,25]. Considering this, the objective of the present study was to determine if FNB-NC encapsulation in NCSMs could be useful in developing improved bioavailability by site-specific release of FNB-NCs.

### 3.1. Preparation of NCSMs

**Figure 3:**
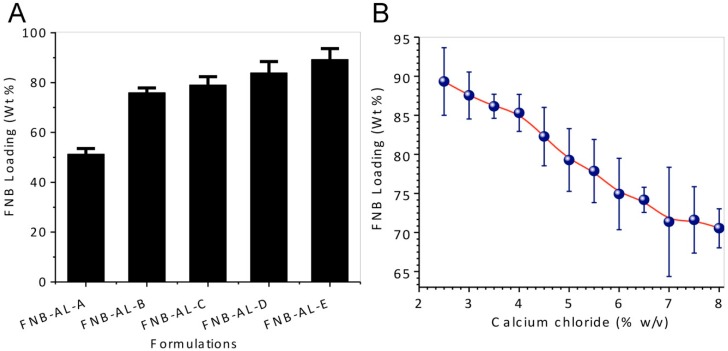
(**A**) Drug-loading capacity of various NCSM formulations, and (**B**) the effect of crosslinker concentration on FNB-NC loading in NCSMs.

Site-specific release of FNB-NCs was obtained by creating NCSMs. We used a simple ionotropic crosslinking technique for the stable and highly effective release of the drug in intestinal pH. The crosslinking reaction between the calcium ions and AL polymeric chains in the aqueous media was responsible for the formation of solid microparticles of the calcium–AL “egg-box” structure [9,19,27,28] with effective encapsulation of FNB-NCs by the gelling process. About 51.34–89.32 wt % of FNB-NCs was encapsulated in NCSMs, depending upon the concentration of the FNB-NC suspensions and crosslinker concentrations (Figure 3A). Furthermore, the FNB-NC loading in NCSMs was highly dependent on the crosslinker concentrations. As the initial concentration of calcium chloride was set at 2.5% (w/v), the amount of FNB-NCs encapsulated in NCSMs was higher and subsequently decreased with higher crosslinker concentrations due to the tight binding of calcium ions with AL polymeric chains (Figure 3B). This is explained by the valency of the binding site in AL polymeric chains that was completely satisfied by calcium ions and the formation of a tight junction between AL polymeric chains making it unavailable for FNB-NCs. It could also be seen visually that semitransparent AL placebo spherical particles were acquired after crosslinking with calcium ions, while off-white NCSMs were obtained due to FNB-NCs in the microparticles. To attain maximum encapsulation of FNB-NCs in NCSMs, the AL:NS ratio was set to 1:16.93 (w/w) and calcium chloride concentration was set to 2.5% (w/v) in the subsequent experiments and further considered for final formulations.

### 3.2. Solid-State Characterization

The solid state of pristine FNB, FNB-NCs, and NCSMs was investigated using XRD, ATR-FTIR, TGA, and DSC techniques. To better investigate these multicomponent formulations, placebo AL spherical particles without FNB-NCs were prepared and used as a reference in this study.

#### 3.2.1. X-ray Diffractograms

**Figure 4:**
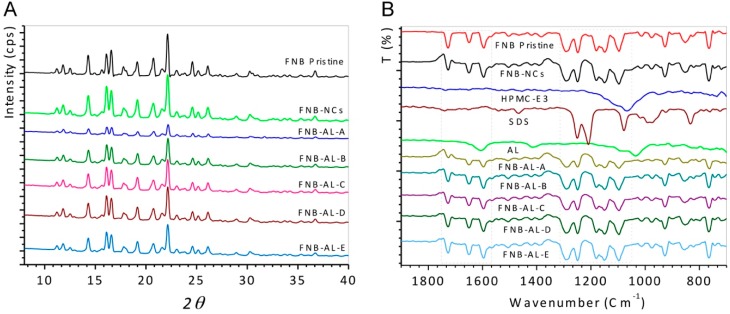
(**A**) X-ray diffraction (XRD) patterns and (**B**) attenuated total reflection Fourier-transform infrared spectroscopy (ATR-FTIR) analysis.

XRD was used to investigate possible changes in the internal structure of drug NCs. Figure 4A shows the XRD spectra of pristine FNB, FNB-NCs, and NCSMs. The typical peaks at a diffraction angle 2θ of 11.8°, 14.3°, 16.10°, 16.60°, 17.75°, 19.20°, 20.80°, 22.10°, and 24.60° were found for pristine FNB, which indicates that they were all in polymorphic crystalline forms. For FNB-NCs and NCSMs, comparable peaks were found but with marginally lesser peak intensities. These results indicate that the crystalline state of FNB-NCs in the NCSMs was unchanged after WSMM processing of FNB, and it also confirmed that the drug particles were well dispersed in the crystalline state in the calcium AL polymer matrix of NCSMs. XRD data also showed that with the increase in FNB-NC loading, the intensities of diffractive peaks for NCSMs also increased accordingly. This is attributed to the change in relative contents of FNB-NCs in the NCSMs. However, the changes in the particle size and the lower restoration of surface charge density during the WSMM process could have caused a minor distinction in the comparative intensities of their peaks.

#### 3.2.2. ATR-FTIR Spectroscopy Analysis

The pristine FNB, FNB-NCs, and NCSMs samples for ATR-FTIR analysis are presented in Figure 4B. Asymmetric and symmetric stretching vibrations of carboxyl anions from AL appeared at 1600 and 1415 cm^−1^ and at 1032 cm^−1^ for the cyclic ether bridge for oxygen stretching [9]. Also, the distinctive peaks of HPMC at 1065 cm^−1^ were recognized as polysaccharide groups (-C-O-C stretching). Peaks from 930 to 1245 cm^−1^ showed -C-O- stretching vibration (AL and HPMC-E3) from NCSMs (all formulations) [29,30,31,32]. The peaks at 1080 and the -SO_2_ head group of SDS were shown at 1210–1250 cm^−1^ for symmetric vibrational and asymmetric vibrational stretching, respectively [33]. The spectrum of pristine FNB and FNB-NCs showed distinctive absorption bands at 1725 cm^−1^, which corresponded to -C=O stretching, and a peak at 1595 cm^−1^, which corresponded to -C=C or -CH_3_ stretching bending. The strong absorption peak at 1270–1295 cm^−1^ was due to -C=O stretching and -OH bending, while the peak at 1180 cm^−1^ was due to -C-O-C ring stretching of the drug [34]. Matched to pristine FNB, FNB-NCs, SDS, and HPMC-E3 characteristic peaks, NCSMs (Formulations A–E) also showed the same peaks and positions, indicating the presence of the FNB-NCs inside the AL matrix and stabilized by the HPMC-E3-SDS complex. With the increase in FNB-NC loading in NCSMs, the peaks intensities for FNB-NCs also increased accordingly (FNB-AL-A > FNB-AL-B > FNB-AL-C > FNB-AL-D > FNB-AL-E). This is recognized to be due to the change in relative contents of FNB-NCs in NCSMs. Overall, there were very few differences between the pristine and FNB-NC spectra, which are most likely to exhibit similar peak positions and intensities with well-defined peaks. These ATR-FTIR spectroscopy studies confirmed that drug NC involvement occurs between stabilized FNB-NCs and AL.

#### 3.2.3. Thermal Analysis 

**Figure 5:**
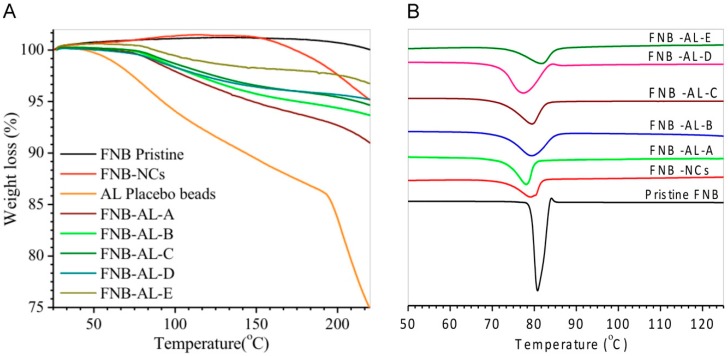
(**A**) Thermogravimetric analysis (TGA) and (**B**) differential scanning calorimetry (DSC) patterns.

Thermal analyses of the pristine FNB, FNB-NCs, and NCSMs are shown in Figure 5A. TGA patterns of AL placebo spherical particles showed a characteristic first-order transition at ~80 °C corresponding to a melting endothermic peak (DSC data) and ~203 °C due to complete decomposition of the AL chain network with calcium ions. The TGA curve of pristine FNB showed single-step weight loss at temperatures of ~200 to 248 °C. This strong endothermic peak was indicative degradation of FNB. The endothermic weight loss peaks of FNB-NCs were shown in three steps at 65, 125, and 246 °C. The first weight loss peak corresponded to the HPMC-E3 melting point (DSC data). This indicated the formation of an HPMC-E3-SDS complex that was present on the surface of the drug particles. From the curves of NCSM weight loss data, it was clearly seen that a marked endothermic weight loss occurred in two distinctive steps at 80–85 and 240–250 °C. This step was directly correlated with AL placebo spherical particles and FNB-NCs. The residual moisture in all formulations are reported in Table 1. All the formulations had a residual of moisture content less than 3.0%, as measured by weight loss in TGA. Apparently, most of the water in the suspensions was removed during the drying process.

DSC spectra showed the thermal behavior of NCSMs (Figure 5B). A lower-temperature sharp endothermic peak of FNB-NCs was shown at 79.25 °C, which is close to the characteristic pattern of the FNB melting temperature [35]. However, NCSMs were exhibited ~80 °C, which corresponded to the melting endothermic peak, the desorption of physically adsorbed water, and the removal of structural water from AL polymeric units and the residue water content from the nanosuspension. FNB-NCs exhibited an endothermic peak with almost double the intensity in the same temperature range. The complete degradation of FNB-NCs and the encapsulated NCSMs probably started above ~200 to 248 °C (TGA data), which was probably due to dehydration of the saccharide rings, as well as infringement of -C-H bonds and C-O-C glycoside bonds in the structural back bone of alginate. No substantial alterations were observed among the FNB-NCs and NCSMs in terms of peak positions and intensities. No additional peaks were found at the lower-temperature scanning range, which indicated that particle aggregation or recrystallization had not occurred during the formulation process, suggesting that no amorphous forms were produced during WSMM and NCSMs. The results suggest that the crystalline structure of FNB was not changed with the milling process. This hypothesis was confirmed by the XRD and ATR-FTIR spectroscopy analysis.

### 3.3. Morphological Characterization

**Figure 6:**
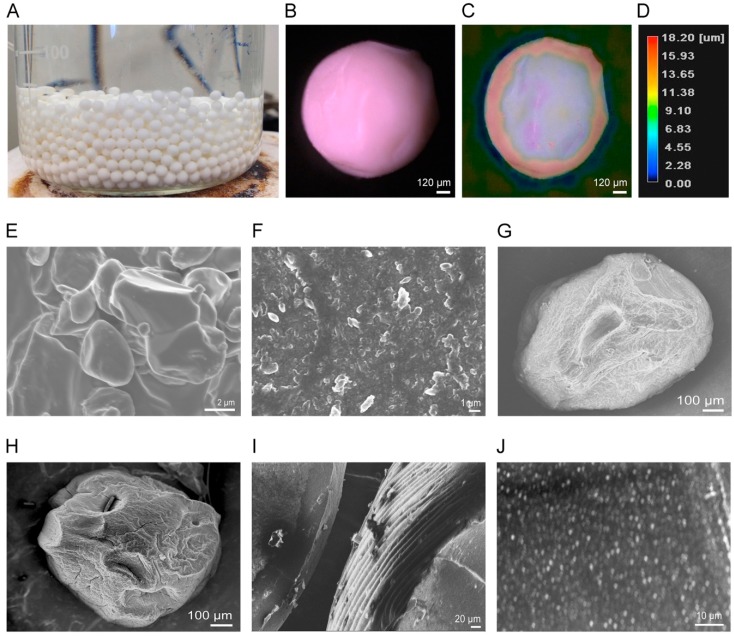
Production of NCSMs via a simple ionotropic crosslinking technique and characterization of the surface morphology. (**A**) Photographic image of microparticles after curing of NCSMs in crosslinker solution. (**B**) Optical microscope image of NCSMs with (**C**) height measurements and (**D**) color code bar. SEM images of (**E**) pristine FNB particles, (**F**) FNB-NCs, (**G**) placebo AL microparticle, (**H**) FNB-NC-encapsulated NCSMs (FNB-AL-E), (**I**) cross section of NCSMs with uniform surface wrinkling patterns, and (**J**) high-resolution cross section of uniformly distributed FNB-NCs in NCSMs.

A photographic image of the NCSMs is shown in Figure 6A. The FNB-NC-loaded NCSMs were spherical in shape and had an average diameter of 1200 ± 250 µm with an even surface (Figure 6B–D) that was confirmed by digital microscopy. To study the surface morphology of the NCSMs samples, SEM was used. The SEM micrographs of the pristine FNB (Figure 6E), FNB-NCs (Figure 6F), and AL placebo microparticle spheres shown in Figure 6G indicate that the spherical particles had wrinkled surfaces. Figure 6E shows that pristine FNB had an uneven shape with a rough surface structure and a particle size of ~10 to 15 µm. Prior to the WSMM process, coarse suspensions of FNB with surfactant solutions consisted of large crystals. After the WSMM process, large FNB-NCs in the presence of the HPMC-E3-SDS stabilizers were transformed into NC form. The angular surfaces of the pristine FNB were much smoother and formed smaller-sized crystals (Figure 6F). While the NCSMs exhibited surface crease patterns identical to the AL placebo microparticles, visual observation indicated that it was milky white and stiffer compared with the AL placebo microparticles due to an interconnection of FNB-NC involvement in the 3D network of calcium AL (Figure 6H). Details of the cross-sectioned surface view of FNB-NC-containing beads are shown in Figure 6I. Furthermore, Figure 6J shows a closer cross-section view of FNB-NCs in the 3D network of calcium AL and homogenized FNB-NCs with minor adherence to the calcium AL polymeric matrix. In parallel with the particle size of coarse suspensions of FNB (~1.80 µm), the particle sizes of FNB-NCs and FNB-NCs in the NCSMs were slightly reduced (~250 to 450 nm). This can be accredited to the high grinding speed in the WSMM process for the FNB coarse suspension preparation. During the WSMM process, shear force and collisions between the drug particles easily fragmented large drug particles into NC particles. The XRD and FTIR analysis results also confirmed that FNB-NCs showed a crystalline structure which was well dispersed within the calcium AL polymeric matrix of NCSMs. 

### 3.4. In Vitro Release Studies

**Figure 7:**
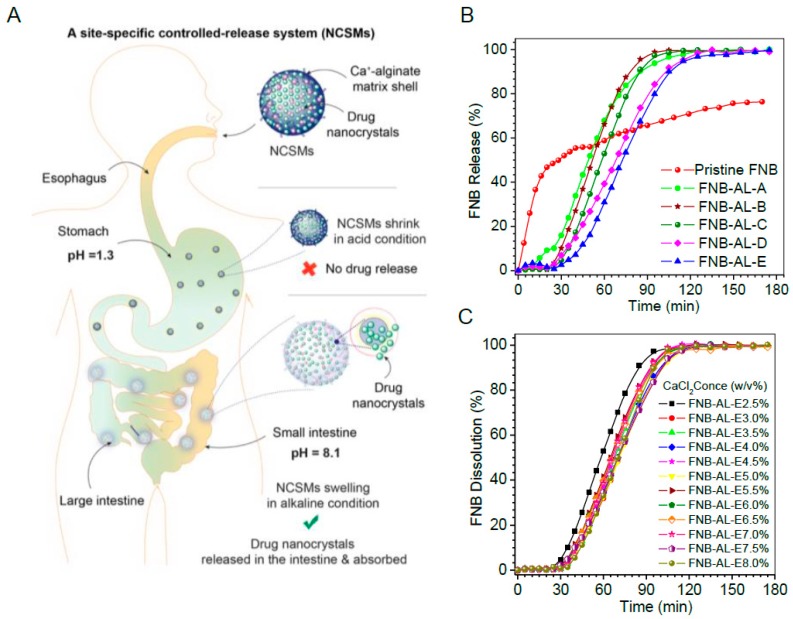
(**A**) A site-specific drug nanocrystal release mechanism of NCSMs. In vitro drug dissolution profile of drug in sodium dodecyl sulfate (SDS) (pH 8.1) with effects of (**B**) drug loading and (**C**) crosslinker concentrations at 37 ± 0.5 °C. Data represent mean ± SD (n = 6). Error bars represent the standard deviation (n = 3) in the Supplementary Figure S1.

Site-specific controlled release behavior of the drug from NCSMs incorporated with different quantities of FNB-NCs and crosslinker concentrations of pH 1.3 (5 mg/mL SDS and pH adjusted with HCl) and pH 8.1 (7.2 mg/mL SDS) is shown in Figure 7. Clearly, the FNB procured from TriCor (Abbott Laboratories, Illinois, IL, USA) was completely released over a short period of time (~12 to 15 min) and only a very small quantity of the drug was released from the NCSM matrix at pH 1.3 (data not shown). In contrast, most of the drug was completely released at pH 8.1. Therefore, the entrapped FNB-NCs released from NCSMs were almost incapable of being released at pH 1.3, while it released in a sustained manner at pH 8.1, making NCSMs an exceptional pH-sensitive matrix for site-specific controlled release, which may be used for oral dosage formulation. The dissolutions of FNB were attributed to swelling of the NCSMs and successful drug rerelease in alkane pH [9]. During the swelling process, anions of SDS from the release media penetrated into the NCSMs with water and reacted with crosslinked calcium alginate. This led to the formation of stable salt bridges between the charged SDS head groups with Ca^2+^ [36]. This SDS–Ca complex created pores within the NCSM structure by removing calcium from the alginate component and releasing FNB-NCs into media. The released NCs were rapidly dissolved and exhibited enhanced bioavailability (Figure 7A). It is also shown in Figure 7B,C that the FNB release patterns were unchanged with the increasing FNB-NC content and crosslinker concentrations from 2.5% to 8% (w/v) (Figure 7C). The fenofibrate drug from TriCor released ~50% within 7 min and ~99.1% within 22 min, while pristine FNB was shown to have an uncompleted dissolution assay. Compared to that from TriCor, all NCSMs were observed to have an initial 25 min lag phase during which only ~2.5% to 3.0% of the drug was released at pH 8.1 (Figure 7B). We assumed that this lag phase of NCSMs was significant for pharmacological action, as it provides time for mucoadhesion and swelling in intestinal mucosa without losing much of the drug. The second phase of the FNB-NC release pattern from NCSMs was shown to be more interesting and had sigmoidal patterns. In this phase, within a short period of ~60 to 90 min, almost the complete release of FNB-NCs from NCSMs occurred at pH 8.1. This result indicates that the FNB-NCs encapsulated in spherical particles could obviously facilitate site-specific modulation of the release and eradicate the burst release hitch of the TriCor drug from the conventional polymeric matrix tablet formulation.

### 3.5. In Situ Real-Time Drug Dissolution Analysis

**Figure 8:**
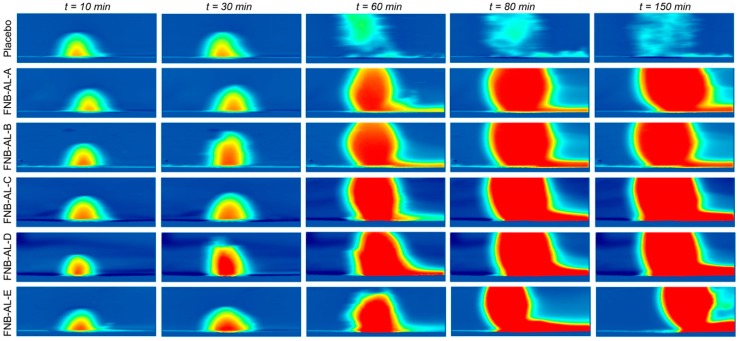
The selective snapshots of real-time UV imaging of FNB-NCs released from NCSM samples under flow conditions (100 µL/min) in SDS media, pH 8.1 (conc. 7.2 mg/mL), at λ_max_ = 280 nm UV absorbance images obtained after 10, 30, 60, 120, and 150 min time intervals.

In nonstop flow conditions, in situ real-time release of the drug from NCSMs was meant to mimic the intestinal environment. The dissolution behavior analyses were conducted using UV-imaging experiments with FNB-NC-containing NCSMs. These NCSMs were mounted on a 3D-printed sample holder and inserted in the UV imager, and the plane to the 3D surface of NCSMs containing the drug was available for picture capture. The ActiPix SDI 300 UV software for the real-time dissolution imaging system calculated the intrinsic dissolution rates (IDRs), surface concentrations, and surface mass release from a 3D downstream gap of a quartz sample cell of the NCSM surface by UV absorbance, which was monitored for 150 min. In NCSMs, the dissolution rate increased after 30–50 min of running the experiments. Selected snapshots illustrate NCSMs containing drug nanocrystal dissolution where the function of time is shown qualitatively (Figure 8). The area in blue shows the background (blank) absorbance, while red, yellow, and light blue show the intensity of the drug concentrations (e.g., deep red color corresponds to elevated concentration (absorbance values)). A short video clip of FNB-NC dissolution from NCSMs can be found in the Supplementary Materials. The photographic inspection of the sample snapshots indicated an improvement of color shade (light blue to dark red) with time. The real-time UV-imaging system was proficient at visualizing the in situ release of drug particles in media observations, which was most likely due to the swelling hydrogel matrix cage of NCSMs.

**Figure 9:**
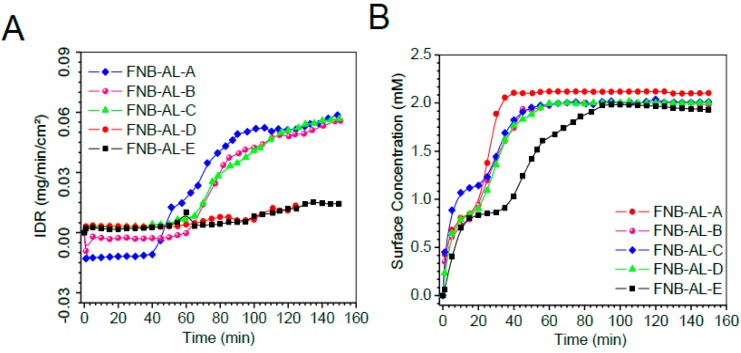
The surface concentration (**A**) intrinsic dissolution rates (IDRs), and (**B**) surface drug concentration of the drug from NCSMs as a function of time obtained by in situ real-time UV imaging at a flow rate of 100 μL/min. Error bars represent the standard deviation (n = 3) in the Supplementary Figure S2.

As displayed in the IDRs in Figure 9A, the dissolution of NCSMs was completed at varying times. Sample mass releases (Supplementary Figure S2C) showed no significant changes with drug contents in NCSMs. Figure 9A shows the surface concentrations of the drug when it swelled in release media followed by FNB-NC release with time, which remained constant after ~40 min. However, Figure 9A displays the IDRs for NCSMs, which showed a direct correlation with the USP-II release pattern. Typically, the highest absorbance contour was collected 40–50 min into the dissolution run for each NCSM samples. In parallel, AL placebo spherical microparticles showed early swelling followed by disappearance due to complete deconstruction of the AL 3D matrix. NC-formulated NCSM samples showed a relative delay with an elevated swelling bump with more red color indicative of UV absorption of the FNB.

## 4. Conclusions

The stabilizer systems of FNB-NCs as suspensions by the HPMC-E3-SDS complex quickly facilitated forming NCSMs using the crosslinking reaction, which provides a new route for transforming FNB-NCs as suspensions into a solid oral dosage as well as overcoming the bioavailability issue by site-specific release. The NCSMs were also shown to have an enhanced drug-loading capacity, which ranged from 50 to 90 wt %. The XRD, DSC, and FTIR analyses suggested that FNB-NCs in the NCSMs remained stable. The FNB-NC dissolution patterns from NCSMs were quantitatively and qualitatively shown by an in situ UV-dissolution imaging system and demonstrated that the release of FNB-NCs is due to swelling followed by diffusion. The overall outcome is that NCSMs containing FNB-NCs can advantageously be used as solid oral dosage forms for site-specific drug release applications. Hence, the established NCSMs conclusively proved site-specific controlled release. However, our NCSMs require further improvement for drying, scaling up for production, and in vivo pharmacokinetics and toxicity characterizations in animal species that have a human-like physiological condition.

## Figures and Tables

**Table 1:** Specifications of starting materials and NCSM formulation parameters.

Formulation Code	AL:NC Ratio (w/w)	Drug Loading (wt %)	NCSM Size (µm) ± SD *	Moisture Content (%)
FNB Pristine	--	100	12 ± 5	-
FNB-NCs	100	90.30	0.28 ± 0.2	-
AL Placebo	--	--	760 ± 50.0	-
FNB-AL-A	1:2.70	51.34	900 ± 80.0	2.6
FNB-AL-B	1:6.77	75.97	1100 ± 130	1.8
FNB-AL-C	1:10.15	79.07	1200 ± 140	1.2
FNB-AL-D	1:13.55	83.94	1200 ± 110	1.7
FNB-AL-E	1:16.93	89.32	1200 ± 250	2.1

* (SD = standard deviation).

## Supplementary Materials

The following are available online at https://www.mdpi.com/1424-8247/12/3/109/s1.
