# Supplementary figures and images for: Fenofibrate Nanocrystal Composite Microparticles for Intestine-Specific Oral Drug Delivery System
Source: Pharmaceuticals (Basel). 2019 Jul 16;12(3):109. doi: 10.3390/ph12030109 (PMC6789785; doi:10.3390/ph12030109)

Figure S1

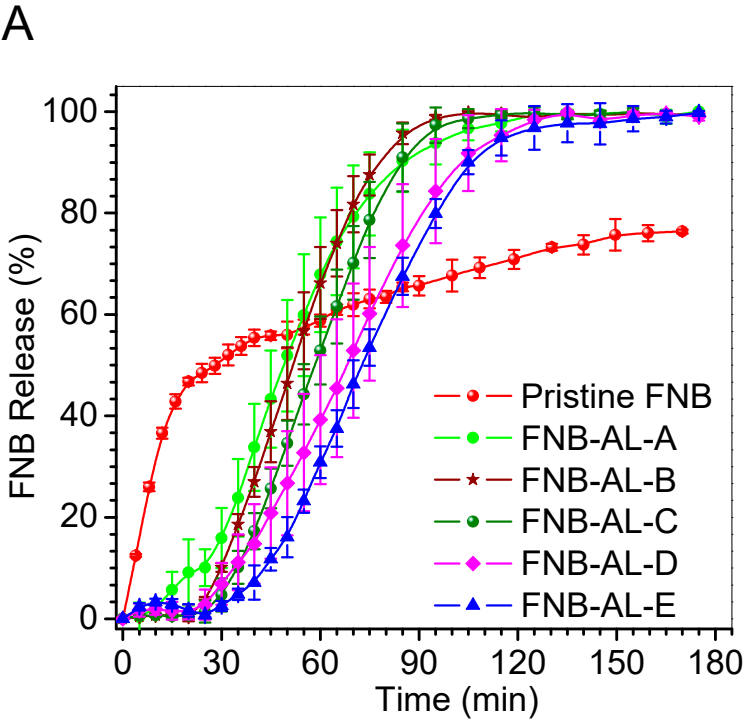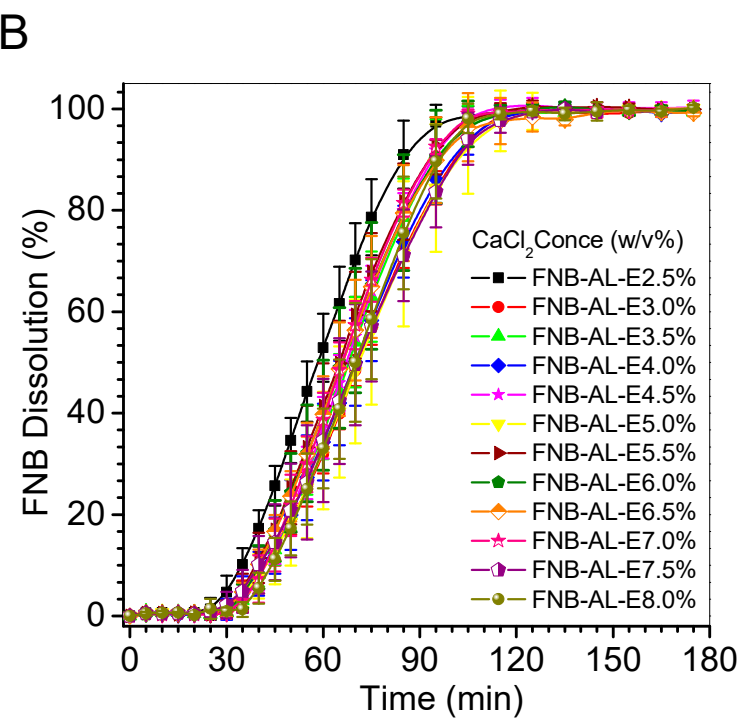

Figure S2

A

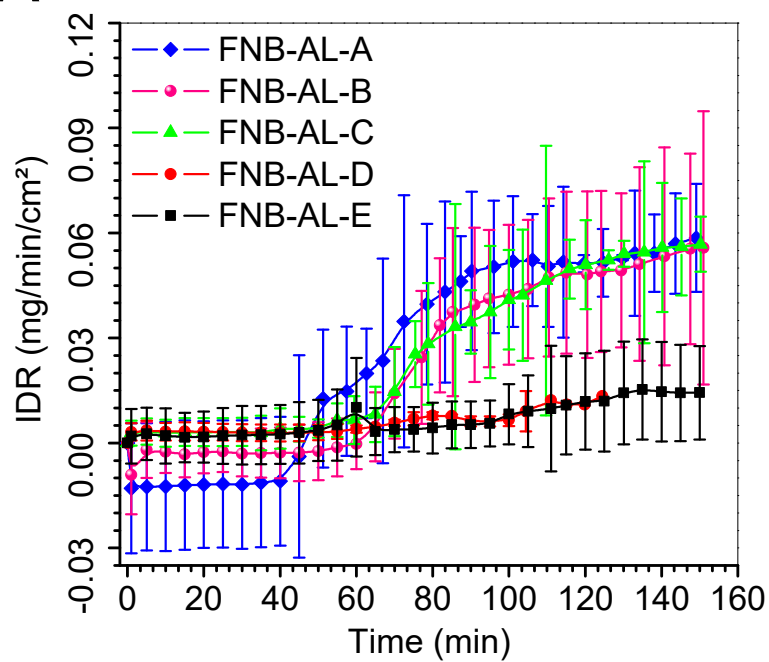

B

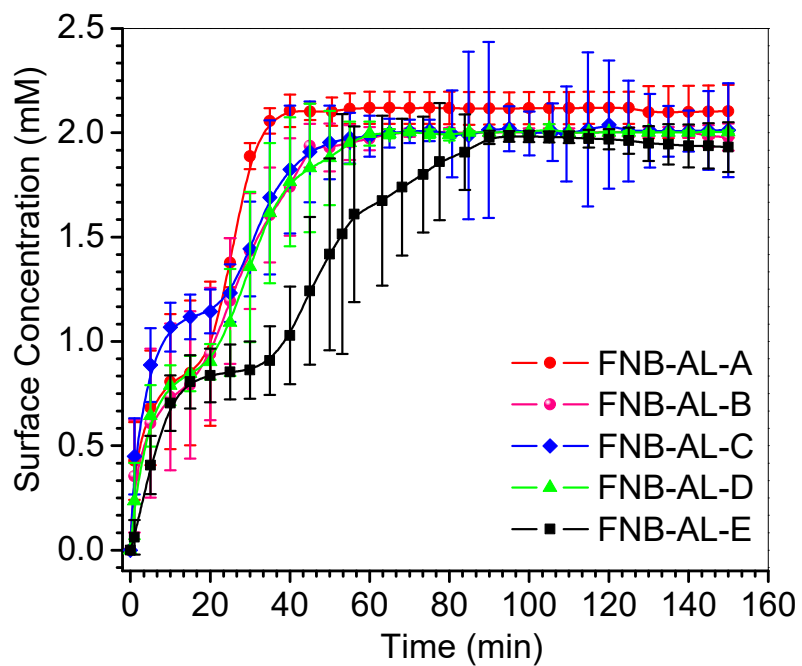

C

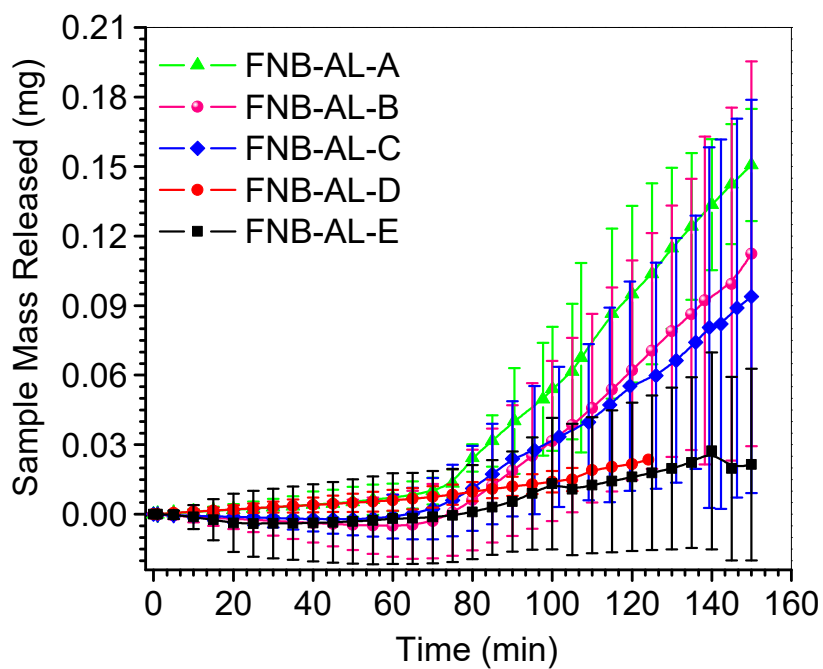

Supplement: Supplementary file 1 [file pharmaceuticals-12-00109-s001.zip › suupl/pharmaceuticals-12-00109-s001-2.pdf]
